# Geographical and age disparities in psychological help-seeking among university students

**DOI:** 10.1007/s44192-026-00415-6

**Published:** 2026-03-09

**Authors:** Patience Araba Mba, Jones Clifford Akosah

**Affiliations:** 1 Gender and Counselling Unit, University of Professional Studies, Accra, Accra, Ghana; 2 College of Psychology, Birmingham City University, Birmingham, UK

**Keywords:** Psychological help-seeking, University students, Ghana, Mental health, Age, Geographical location

## Abstract

This study investigated the influence of age and geographical location on psychological health-seeking behaviour among university students in Ghana, drawing on the Health Belief Model (HBM) and the Theory of Planned Behaviour (TPB) as guiding frameworks. The research targeted 5369 undergraduate accounting students across four public universities, with 588 respondents completing an online survey. Attitudes toward professional psychological help were assessed using the 10-item ATSPPH-SF scale, which demonstrated acceptable internal consistency in this context. Data were analysed using ANOVA and independent-samples t-tests, with effect sizes and confidence intervals reported. The results showed no significant differences in health-seeking behaviour across age groups. However, geographical location was found to be a significant determinant, as students from urban areas were more likely to seek professional psychological support compared with their rural counterparts. Consistent with the HBM, rural students may perceive greater barriers and lower benefits in accessing care, while TPB suggests that normative pressures and perceived behavioural control influence students’ willingness to seek help. These findings highlight the need for targeted interventions to address structural and cultural barriers in rural settings. Expanding mental health accessibility across diverse geographical areas should be prioritized by universities and policymakers.

**Supplementary Information:**

The online version contains supplementary material available at 10.1007/s44192-026-00415-6.

## Introduction

Health-seeking behaviour refers to any action an individual undertakes in response to a perceived health concern [42]. Such actions may include self-medication, seeking treatment from traditional or modern healthcare providers, or, in some cases, forgoing care altogether [14, 48]. A variety of factors influence these behaviours, including the type and severity of illness, gender, sociocultural context, treatment costs, cultural beliefs about illness, quality of care, education, and financial resources [25].

Mental health challenges among young adults are increasingly common, particularly in higher education settings. Since the COVID-19 pandemic, the prevalence of psychological distress among students has risen sharply [40]. For instance, Rahman et al. [40] found high levels of depression, anxiety, and stress among Bangladeshi university students, while Zurlo et al. [50] reported similar patterns in Italy. In Ghana, students face comparable psychological difficulties, including stress, depression, anxiety, and suicidal ideation. Alarmingly, the Kintampo Health Research Centre [29] reported two student suicides in 2019, underscoring the urgency of addressing these issues.

Despite these challenges, many students display negative attitudes toward seeking counselling or psychological support. Health-seeking behaviour is a multidimensional process shaped by an individual’s circumstances and the duration of their health condition. Psychological health-seeking behaviour, therefore, refers specifically to the actions individuals take when experiencing mental health difficulties, including their willingness to seek professional help. Such behaviour is influenced by factors such as age, geographical location, socioeconomic status, cultural beliefs, and perceived stigma [39]. While most research on psychological help-seeking behaviour has been conducted in high-income countries, there is growing recognition of the need to investigate these factors in Ghana, where stigma, literacy, and access to services vary considerably [8]. For example, Joseph and Edward [28] found that many Ghanaian students held negative attitudes toward counselling services despite their availability, a concern given that untreated distress can contribute to social withdrawal, academic difficulties, and even suicide [27, 33].

Studies from sub-Saharan Africa provide mixed evidence. Research in Botswana [3] and Ethiopia [3] revealed conflicting findings on students’ attitudes toward help-seeking, but neither examined underlying determinants. In Ghana, research remains scarce, with Andoh-Arthur et al. [8] offering the only published study to date. While insightful, their work was limited to students within a single university department, restricting its generalizability.

This study draws on the Health Belief Model (HBM) [41] and the Theory of Planned Behaviour (TPB) [2] to examine psychological help-seeking behaviour among Ghanaian students. The HBM posits that individuals are more likely to seek care if they perceive themselves as vulnerable to illness, recognize its severity, believe in the benefits of treatment, and face minimal barriers to access. The TPB emphasizes attitudes toward seeking help, social norms, and perceived behavioural control as predictors of behaviour.

Guided by these frameworks, this study investigates whether age and geographical location influence students’ psychological help-seeking behaviour. Older students may have greater awareness and less stigma, while younger students may struggle with identifying and managing emotions [43]. Similarly, urban students may benefit from greater service availability, whereas rural students often face stigma, financial limitations, and shortages of trained professionals [46].

By integrating these theoretical perspectives, this research aims to clarify how age and geographical location affect help-seeking behaviour among Ghanaian university students. Insights from this study will be valuable for developing interventions that improve mental health awareness, reduce stigma, and expand access to services across higher education institutions in Ghana.

## Review of related literature

This section reviews the literature on age and geographical location as determinants of psychological health-seeking behaviour. Drawing on previous studies, hypotheses are generated.

### Age and health-seeking behaviour

Age is a critical determinant of health-seeking behaviour, but its role is not uniform across contexts. Evidence from recent global studies suggests that age can act both as a facilitator and a barrier, depending on sociocultural, economic, and structural conditions.

On the one hand, age facilitates health-seeking when older adults possess resources or supports that encourage engagement with healthcare. In Ethiopia, for example, older individuals with higher education, family support, and community-based health insurance were significantly more likely to seek care, particularly in the presence of chronic illness [12]. Similarly, in Singapore, older adults with greater digital literacy were more engaged with health services, underscoring how technological competence can enhance access for ageing populations [16]. In Turkey, older university students reported more positive attitudes toward psychological help-seeking than their younger peers, suggesting that maturity and emotional awareness can encourage service utilisation [44].

On the other hand, age can also serve as a barrier. In Saudi Arabia, younger women were more proactive in seeking care compared with older women, whose reliance on cultural beliefs, low perceived severity of symptoms, and limited awareness delayed healthcare use [4]. In India, older adults were less likely to seek treatment for non-communicable diseases, with barriers including financial constraints, lack of awareness, and reliance on traditional remedies [22]. Similar challenges were reported in Ghana, where even insured older adults often delayed care due to transportation costs and gaps in coverage, sometimes resorting to unprescribed medication [13, 20, 32].

For younger populations, the evidence is also mixed. In Zambia, adolescents (15–19 years) were more likely than adults to use healthcare services, possibly due to heightened health concerns during this life stage [51]. Yet, in South Africa, adolescents were less likely to seek care consistently, often relying on local clinics rather than formal healthcare systems [36].

Taken together, these findings show that age operates in complex ways: it can facilitate health-seeking when coupled with education, social support, and access to resources, but it can also hinder utilisation when structural, cultural, or financial barriers outweigh these advantages. The present study extends this literature by examining health-seeking behaviour in a younger Ghanaian student population—a group often overlooked despite their vulnerability to financial and cultural barriers [44, 47].

Building on the reviewed evidence, the following research question and hypotheses are proposed:

**Research Question 1:** How does age influence psychological health-seeking behaviour among university students in Ghana?

- *H01*: There is no significant age variation in the psychological health‐seeking behaviour of public university students.
- *HA1*: There is a significant age variation in the psychological health‐seeking behaviour of public university students.

### Geographical location and health-seeking behaviour

In this study, “geographical location” refers to students’ permanent place of residence during vacation periods rather than the location of their university. This approach captures long-term exposure to healthcare environments rather than the temporary influence of the university setting. Following the Ghana Statistical Service [19], students were classified as urban (residing in localities with a population of 5,000 or more) or rural (less than 5,000). This classification reflects differences in access to mental health facilities, availability of professionals, and community awareness of psychological services.

Geographical background plays a central role in shaping health-seeking behaviour, as access, affordability, and stigma vary between rural and urban settings. Rural communities often face shortages of trained professionals, long distances to health facilities, and financial or infrastructural barriers, all of which contribute to underutilization of mental health services [4, 9, 49]. These disparities persist even where services exist, as shown in the USA and Turkey, where rural residents frequently delay seeking care or rely on informal support due to systemic and logistical constraints [7, 35]. Research in India indicated that rural individuals were less likely to seek treatment for non-communicable diseases due to financial constraints, low awareness, and dependence on traditional remedies [21]. Evidence from South Africa during the COVID-19 pandemic revealed significant regional variations in healthcare utilisation, indicating that local infrastructure and resource availability are critical determinants of health-seeking behaviour [6].

Similar patterns are evident in Ghana and across sub-Saharan Africa. Amegbor [5] found that while both rural and urban residents in the Asikuma-Odoben-Brakwa District frequently engaged in self-medication, formal help-seeking was more common among those with higher education. Afeadie [1] reported that slum dwellers in Accra were constrained by financial hardship and poor living conditions, often turning to non-professional sources of care. Perreira [37] further identified transport costs, health facility capacity, and long travel times as barriers discouraging timely healthcare utilization in rural Ghana.

Although some studies, such as Idriss et al. [26] in Sierra Leone, suggest comparable levels of health-seeking knowledge between rural and urban residents, the broader evidence highlights persistent disadvantages faced by rural populations in accessing psychological services. These disparities justify the need to examine whether Ghanaian university students from rural and urban backgrounds differ in their psychological help-seeking attitudes.

Based on the reviewed literature, the following research question and hypotheses are proposed:

**Research Question 2:** What role does geographical location play in shaping students’ attitudes toward psychological help-seeking?

- *H01:* There is no significant geographical variation in the psychological health seeking behaviour of public university students.
- *HA1:* There is a significant geographical variation in the psychological health‐seeking behaviour of public university students.

**Fig. 1:**
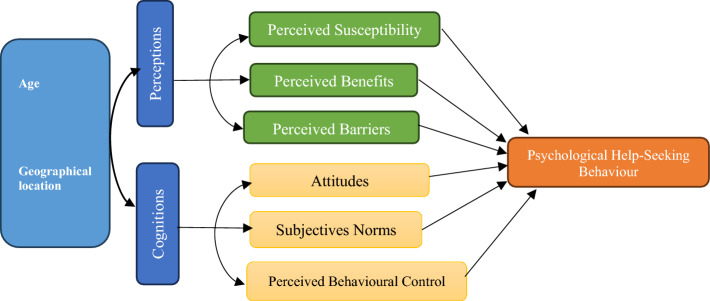
Conceptual framework linking age, geography, HBM/TPB constructs and help-seeking behaviour

The conceptual framework figure visually links age and geographical location to the core constructs of the Health Belief Model (HBM) and Theory of Planned Behaviour (TPB), showing how these ultimately shape students’ psychological help-seeking behaviour. It demonstrates how demographic factors (age, location) influence perceptions (susceptibility, benefits, barriers) and cognitions (attitudes, norms, control), which in turn determine the likelihood of seeking professional psychological help (Fig. 1).

## Methodology

### Study design and population

This study employed a cross-sectional survey design to collect data from undergraduate students at four selected public universities in Ghana: University of Professional Studies (UPSA) in the Greater Accra Region, Kwame Nkrumah University of Science and Technology (KNUST) in the Ashanti Region, University for Development Studies (UDS) in the Northern Region, and University of Cape Coast (UCC) in the Central Region. These universities were purposefully selected to provide geographic representation across Ghana, encompassing the coastal belt, forest zone, and savannah areas. Each institution maintains well-equipped counselling centres accredited by the Ghana Psychological Council, a resource not consistently available in many private tertiary institutions. The study targeted a population of 5,369 students enrolled in Business programmes with an Accounting option, chosen because this programme is offered across all four universities.

### Participants

The sampling frame included 5,369 undergraduate Business students (Accounting option) across UPSA, KNUST, UDS, and UCC. Data were collected via an online survey administered through Google Forms, distributed on class and group platforms between 10 December 2020 and 30 March 2021. Of those who accessed the survey, 588 students provided complete responses, yielding an overall response rate of 11.0% (588/5369).

Sample size estimation followed Krejcie and Morgan’s [30] guidelines. For a population of 5,000, the minimum recommended sample size is 537, which was rounded up to 588 to enhance external validity. Krejcie and Morgan’s formula was applied in the calculation to ensure adequate representation of the target population.

$$ s = \frac{{X^{2} NP\left( {1 - P} \right)}}{{d^{2} \left( {N - 1} \right) + X^{2} P\left( {1 - P} \right)}}\,{\mathrm{where}}, $$

s = required sample size. X^2^ = the table value of chi-square for 1 degree of freedom at the desired confidence level (3.841). N = the population of business students in the selected four public universities. P = the population proportion (assumed to be 0.50 since this would provide the maximum sample size). d = the degree of accuracy expressed as a proportion (0.05).

This estimated sample size was rounded up to 550, as increasing the sample size enhances the credibility of the results [16].

A multistage sampling procedure employing purposive, simple random, and proportional stratified sampling techniques was utilized. In the first phase, purposive sampling was used to select the four public universities across the country, as well as students enrolled in Business programmes with an accounting option. In the second phase, proportional stratified sampling was employed to determine a proportionate number of respondents based on gender and academic level at each university, using the formula below:

$$ \frac{{\text{Sample size}}}{{{\text{Total }}\,{\mathrm{Population}}}} \times {\mathrm{Total}}\,{\text{ number }}\,{\text{of }}\,{\mathrm{males}}/{\mathrm{females}}\,{\text{ for }}\,{\text{each }}\,{\mathrm{level}} $$

In the third phase, a convenience sampling approach was used due to the constraints of online data collection. This involved selecting students who were available and willing to participate. This approach was necessary because of the COVID-19 pandemic, which meant that only a few students were accessible, as the majority were attending online lectures from home. While this method ensured efficient data gathering, it may have introduced potential biases, since students with limited internet access or lower digital literacy might have been underrepresented.

Table 1 shows the distribution of the sample according to academic level and gender.

**Table 1:** Distribution of sample by academic level and gender

Level	KNUST			UCC			UDS			UPSA		
	M	F	Total	M	F	Total	M	F	Total	M	F	Total
100	22	18	40	6	6	12	19	6	25	25	20	45
200	20	15	35	8	7	15	20	7	27	22	17	39
300	30	21	51	7	6	13	21	7	28	36	27	63
400	36	29	65	4	4	8	19	6	25	33	26	59
Total	108	83	191	25	23	48	79	26	105	116	90	206

From Table 3, the students have been grouped into strata, namely gender and levels for each university. Under each stratum, a proportional allocation was used to select a sample size.

### Data collection

Data were collected using an online questionnaire due to COVID-19 restrictions during the study period. The questionnaire was created using Google Forms and distributed through students’ class group platforms across UPSA, KNUST, UDS, and UCC. This online approach allowed for broader reach and convenience, enabling students to participate regardless of their physical location.

The questionnaire link included clear instructions regarding participation, anonymity, and informed consent to ensure ethical compliance. Some students completed the survey from home during ongoing online lectures, while others responded on campus once in-person classes resumed. To encourage participation, students were provided with mobile airtime valued at ₵5 upon completion, as described in the participant information sheet.

A total of 588 completed responses were received and used. The questionnaire included demographic items and the Attitudes Toward Seeking Professional Psychological Help–Short Form (ATSPPH–SF) scale.

### Measures

The ATSPPH-SF [18] (10 items), using the revision by Heaslip [22], was adopted to assess student attitudes toward seeking professional psychological help. The instrument was reviewed by two Ghanaian counselling academics for face validity and piloted with 60 students (excluded from the analytic sample) to assess clarity,minor wording adjustments were made for comprehension. No translation was required. Each item was scored from 1 to 4 and summed (range 10–40), with scores ≥ 25 indicating a favourable attitude. Internal consistency was acceptable, with Cronbach’s α = 0.70 in both the pilot and analytic samples (95% CI 0.66–0.74). The scale demonstrated similar reliability in prior studies (α = 0.73) [18, 31].

The choice of the ATSPPH-SF instrument was theoretically grounded in the Health Belief Model (HBM) and the Theory of Planned Behaviour (TPB). The HBM highlights the role of perceived susceptibility, perceived benefits, and perceived barriers in shaping health-related decisions. These constructs align with items in the ATSPPH-SF that assess students’ recognition of the usefulness of professional help and their concerns about stigma or costs. Similarly, the TPB emphasizes attitudes, subjective norms, and perceived behavioural control, all of which are reflected in the instrument’s focus on students’ willingness, social comfort, and perceived ability to seek psychological help. Thus, the instrument was not only psychometrically reliable but also theoretically consistent with frameworks that explain help-seeking behaviours.

### Ethical considerations

Ethical approval was obtained from the Institutional Review Board of the University of Cape Coast (Approval Number: UCCIRB/CES/2020/88). Electronic informed consent was obtained on the first page of the Google Form, and participants had to confirm consent before proceeding. Responses were collected anonymously and stored securely on the lead author’s institutional account. Airtime incentives were delivered without linking participants’ identities to their responses; mobile numbers provided for incentive delivery were stored separately and destroyed after use. All methods were carried out in accordance with relevant guidelines and regulations.

### Statistical analysis

Analyses were conducted in SPSS v24. The questionnaires were carefully reviewed and edited to ensure accuracy and consistency of the data. We examined distributions and used Levene’s test for equality of variances. For group comparisons, we report means ± SD, t-tests, ANOVA with F(df between, df within). For t-tests we report Cohen’s d with 95% Cis. A two-sided α = 0.05 significance threshold was used. All code for data cleaning and analysis is available at upon request.

## Results

Compared with the sampling frame, respondents were over-represented by second year, male, urban and 18–24-year-old students, suggesting potential non-response bias which may limit generalisability.

### Demographic characteristics of respondents

**Table:** 

Variables	Category	Frequency	Percent
University	UPSA	216	36.7
	UCC	71	12.1
	KNUST	179	30.4
	UDS	122	20.8
	Total	588	100.0
Gender	Male	305	51.9
	Female	283	48.1
	Total	588	100.0
Age groups	18–24	485	82.5
	25–29	79	13.4
	30+	24	4.1
	Total	588	100.0
Geographical	Urban	392	66.7
Location	Rural	196	33.3
	Total	588	100.0
Academic level	100	111	18.9
	200	203	34.5
	300	146	24.8
	400	128	21.8
	Total	588	100.0

### Age and psychological health-seeking behaviours

Understanding health behaviour of university students could also open important venues of intervention. For example, health behaviour of students that is linked to “powerful others” health beliefs (PLOC) might be of interest for health campaigns.

H_0_: There is no association between locus of control and psychological health-seeking behaviour of public university students in GhanH_1_: There is an association between locus of control and psychological health-seeking behaviour of public university students in Ghana.

This section presents the analysis of the impact of age on psychological help-seeking behaviour. Respondents were grouped into three age categories: 18–24, 25–29, and 30 years and above. A one-way ANOVA was conducted to determine whether there were statistically significant differences in psychological help-seeking scores across these age groups. The result is presented in Table 2.

**Table 2:** ANOVA tests for psychological health-seeking behaviours based on age

Variables	Source of variations	Sum of squares	df	Mean square	F	*p*-value
Psychological Health-seeking behaviour	Between Groups	5.86	2	2.93	0.224	0.799
	Within groups	7653.97	585	13.08		
	Total	7659.83	587			

*Key*: t—*t-statistic* (used in t-tests to compare means); df—*degrees of freedom* (reflects the number of independent values that can vary in a calculation); N—*sample size* (the total number of participants in a study); F—*F-statistic* (used in ANOVA or regression to test for differences among group means)

The results indicate no significant difference in psychological help-seeking behaviour among the three age groups, F(2, 585) = 0.224, *p* = 0.799. Since the p-value exceeds the 0.05 threshold, the null hypothesis is accepted. This suggests that age does not significantly influence the psychological help-seeking behaviour of university students in Ghana.

### Geographical location and psychological health-seeking behaviours

To assess whether students’ place of residence (urban or rural) influenced their psychological help-seeking behaviour, an independent samples t-test was conducted. Students were grouped based on their responses to a survey question regarding their permanent residence outside of the university setting. The result is presented in Table 3.

**Table 3:** Independent samples t-test of psychological health-seeking behaviours based on geographical location

	N	Mean	SD	t	Df	Sig. (2-tailed)
Rural	392	27.34	3.71			
				− 2.991*	586	0.003
Urban	196	28.28	3.33			

^*^Significant, *p* < 0.05

The Levene’s test for equality of variances for the two variables tested produced a p-value greater than 0.05. This means that at 5% level of significance, there is an equal variability between respondents’ geographical location (urban and rural) scores for psychological health-seeking variables. Therefore, equal variances assumption can be held. Students from Urban backgrounds (N = 196) had a higher mean ATSPPH-SF score (M = 28.28, SD = 3.33) than students from rural backgrounds (N = 392; M = 27.34, SD = 3.71). Independent-samples t-test: t(586) = − 2.991, *p* = 0.003; Cohen’s d = 0.26 (95% CI 0.09–0.43), indicating a small effect in favour of urban-origin students.

## Discussion

### Age and psychological health-seeking behaviour

This study examined the relationship between age and psychological health-seeking behaviour among university students. The results indicate no significant differences in mean scores across the three age groups, suggesting that both younger and older students hold similar attitudes toward seeking psychological support. This finding aligns with Arku [10], whose study of male students at the University of Cape Coast (UCC) found that age did not significantly influence attitudes toward psychological help-seeking. Although Arku’s sample was limited to 345 male students, the results are broadly consistent with those of the present study, which included a larger and more diverse cohort.

In contrast, research in Zambia by Zyaambo et al. [51] showed that urban residents over 30 were twice as likely to use healthcare facilities as those aged 15–19. Similarly, Otwombe et al. [36] observed lower healthcare utilization among adolescents in South Africa. These studies suggest that older individuals may possess greater experience and awareness regarding their health, and, as adults, they may have more financial resources, facilitating positive health-seeking behaviours.

Seyfi et al. [43] reported that older Turkish university students exhibited more favourable attitudes toward psychological help-seeking than their younger counterparts, attributing the differences to younger students’ difficulty in identifying and expressing emotions and to challenges associated with transitioning to university life. The discrepancy with the current study may be due to Seyfi et al. inclusion of graduate students, which increased the proportion of older respondents.

Empirical evidence from sub-Saharan Africa and Europe indicates that age can influence health outcomes, with older individuals generally experiencing poorer health. These mixed findings suggest inconsistencies in the literature regarding the role of age in shaping health-seeking attitudes: some studies report better attitudes among older adults [51], whereas others indicate more positive behaviours among younger individuals [11].

The findings of this study can be more deeply understood through the lenses of the HBM and TPB. The absence of significant age differences in help-seeking behaviour suggests that individual beliefs and social influences, rather than chronological age, may be more decisive in shaping students’ attitudes. This interpretation is consistent with the TPB, which posits that behaviour is determined by attitudes, perceived norms, and behavioural control.

### Geographical location and psychological health-seeking behaviour

Geographical location emerged as a significant determinant of psychological health-seeking behaviour in this study. Conceptualized as students’ place of residence during vacation periods, this variable captures long-term exposure to local healthcare environments. Independent-samples t-tests revealed that students from urban areas exhibited significantly more positive health-seeking behaviours than those from rural backgrounds. Although some rural-origin students attend universities in urban centres, exposure to urban lifestyles does not appear to fully mitigate differences in attitudes toward professional psychological support.

Several factors may explain these disparities. Rural students often come from families with lower socio-economic status, which can limit access to professional healthcare. Additionally, parental health-seeking behaviours and reliance on traditional or informal care may influence students’ attitudes. The geographic distribution of psychological healthcare facilities, which are predominantly located in urban areas, further contributes to these differences by imposing additional transportation costs and travel time for rural students.

Previous studies support these findings. Van der Hoeven et al. [46] reported that urban residents generally have broader access to services, including private facilities, a pattern that mirrors the socio-economic disparities observed among Ghanaian university students. Yikilkan et al. [49] found that rural residents in Turkey faced long distances, inadequate facilities, and extended waiting times, resulting in less positive health-seeking behaviours an observation that parallels the present findings. Afeadie [1] similarly highlighted the role of financial constraints in driving rural residents to rely on informal care, while Perreira [37] documented the impact of travel distance on healthcare utilization in rural Ghana.

Conversely, Idriss et al. [26] in Sierra Leone found comparable health-seeking behaviours among rural and urban populations, suggesting that context-specific factors such as the uneven distribution of psychological services in Ghana may explain differences observed among university students.

The significant role of geographical location resonates with the HBM: students in rural areas may perceive greater barriers (e.g., stigma, transportation, cost) and fewer benefits in accessing psychological services, while urban students experience more enabling environments. By integrating these models, the study highlights that students’ decisions to seek psychological help are not random but shaped by structured beliefs, perceptions, and social contexts.

Overall, the findings underscore the influence of geographical background on attitudes toward psychological healthcare, highlighting the persistent challenges faced by rural-origin students in accessing professional mental health services, even when studying in urban universities. These results have implications for interventions aimed at reducing disparities in psychological health-seeking behaviour across geographic settings.

## Limitations and directions for further research

While this study provides valuable insights, several limitations should be acknowledged. The cross-sectional design precludes causal inferences. The lack of qualitative data is another limitation which could have added depth to the study. The online convenience sampling method, combined with a relatively low response rate (~ 11%), may have introduced selection bias, potentially over-representing students with internet access or an interest in mental health. The provision of airtime incentives could have influenced participation patterns. Additionally, measures were self-reported and therefore susceptible to social desirability bias. Data on socioeconomic status and prior contact with mental health services were not collected, representing potential confounding variables.

Another key limitation is the focus on undergraduate students enrolled specifically in Business programmes (Accounting option). While this approach provided a homogeneous sample and was feasible during COVID-19 restrictions, it limits the generalizability of the findings to students from other academic disciplines who may experience different stressors, support systems, and attitudes toward help-seeking. Future research should therefore consider using stratified sampling across multiple faculties and programmes to capture the diverse academic and social contexts of Ghanaian university students, thereby improving the external validity of findings.

Despite these limitations, the study makes a significant contribution to the limited research on psychological help-seeking behaviour among university students in Ghana, providing a foundation for future research and policy interventions. Future studies could adopt longitudinal designs, incorporate more diverse sampling methods, and include variables such as socioeconomic status, prior service use, and cultural perceptions to provide a more comprehensive understanding of help-seeking behaviour.

### Implications of the study

The findings have important implications for mental health professionals, university counselling centres, and policymakers aiming to enhance psychological help-seeking behaviour among Ghanaian students. First, the absence of significant age differences suggests that interventions should target all students, with counsellors adopting developmentally appropriate approaches that cater to both younger and older cohorts.

Second, the significant impact of geographical location highlights the need for targeted support for rural-background students. Universities should consider mobile mental health services, virtual counselling platforms, and satellite counselling offices to improve access. Culturally sensitive psychoeducation and engagement with community, religious, and traditional leaders through workshops can help reduce stigma.

Third, bridging the accessibility gap for rural students may require financial aid, integration of counselling services into student health insurance, and peer-led mental health advocacy to normalize help-seeking. Counsellor training and service delivery should be culturally responsive, reflecting students’ social, religious, and traditional contexts. Hiring counsellors from diverse backgrounds, including those with rural experiences, may also foster trust and engagement.

Finally, universities should strengthen counselling units by increasing counsellor-student ratios, extending service hours, integrating mental health support into academic advising, and implementing proactive screening, workshops on stress management, and early intervention programs. Such measures can shift help-seeking from reactive crisis management to preventive care, creating a more inclusive and supportive environment.

## Conclusion

This study evaluated the influence of age and geographical location on the psychological help-seeking behaviour of Ghanaian university students. Findings indicate that age does not significantly affect attitudes toward psychological healthcare, with students across age groups demonstrating similar behaviours. In contrast, geographical location plays a significant role, with urban-background students exhibiting more positive help-seeking behaviours than their rural counterparts. These results underscore the importance of considering geographical disparities when designing interventions and programs to promote mental health support and counselling services among Ghanaian students.

## Supplementary Information

Below is the link to the electronic supplementary material.

Supplementary Material 1

## Data Availability

The datasets used during the current study are available from the first author on reasonable request.
