# Supplementary material for: Geographical and age disparities in psychological help-seeking among university students
Source: Discov Ment Health. 2026 Mar 9;6(1):63. doi: 10.1007/s44192-026-00415-6 (PMC13087063; doi:10.1007/s44192-026-00415-6)
Supplement: Supplementary file 1 — Supplementary Material 1 [file 44192_2026_415_MOESM1_ESM.docx]

**Attitudes Toward Seeking Professional Psychological Help Scale Short-Form (ATSPPHS-SF)**

**Instruction**: Tick [√] one of the responses for all the statements and do not leave any blank. The response options are Strongly Agree (SA); Agree (A); Disagree (D); Strongly Disagree (SD).

| **No.** | **STATEMENT** | **SA** | **A** | **D** | **SD** |
| --- | --- | --- | --- | --- | --- |
| 1. | If I believed I was having a mental breakdown, my first inclination would be to get professional attention. |  |  |  |  |
| 2. | The idea of talking about problems with a psychologist strikes me as a poor way to get rid of emotional conflicts. |  |  |  |  |
| 3. | If I were experiencing a serious emotional crisis at this point in my life, I would be confident that I could find relief in psychotherapy. |  |  |  |  |
| 4. | There is something admirable in the attitude of a person who is willing to cope with his or her conflicts and fears without resorting to professional help. |  |  |  |  |
| 5. | I would want to get psychological help if I were worried or upset for a long period of time. |  |  |  |  |
| 6. | I might want to have psychological counseling in the future. |  |  |  |  |
| 7. | A person with an emotional problem is not likely to solve it alone; he or she is likely to solve it with professional help. |  |  |  |  |
| 8. | Considering the time and expense involved in psychotherapy, it would have doubtful value for a person like me. |  |  |  |  |
| 9. | A person should work out his or her own problems; getting psychological counselling would be a last resort. |  |  |  |  |
| 10. | Personal and emotional troubles, like many things, tend to work out by themselves. |  |  |  |  |
